# Hashimoto Thyroiditis Coexisting With Breast Fibroadenoma

**DOI:** 10.7759/cureus.53585

**Published:** 2024-02-04

**Authors:** Arushi Arvind, Walaa Omer, Alaa Ahmed Mahdi

**Affiliations:** 1 Department of Internal Medicine-Endocrinology, HMS Al Garhoud Hospital, Dubai, ARE

**Keywords:** autoimmune, thyroid pathology, general internal medicine, benign breast condition, hashimoto’s thyroiditis

## Abstract

A 34-year-old female with co-existing multiple breast nodules being treated simultaneously presented to the outpatient clinic with fatigue as the chief complaint which had progressively worsened over one year. Ultrasound showed a heterogeneous parenchymal ectopic pattern suggestive of thyroiditis, with no suspicion of nodules and cysts. Laboratory results showed raised levels of thyroid-stimulating hormone (TSH), serum anti-thyroglobulin antibody, and serum thyroid peroxidase antibody. Levothyroxine sodium at a dosage of 50 µg/day was prescribed to the patient, following which the patient had normal TSH levels on follow-up after two months. Simultaneously the patient was under investigation for the breast nodules that were seen as the patient’s medical history when she presented to the endocrinology clinic. She was diagnosed with fibroadenoma with a canalicular pattern, without ductal atypia in both breasts and malignancy.

## Introduction

One of the most common endocrine disorders globally is hypothyroidism which is defined as the inadequate production of the thyroxine (T4) and triiodothyronine (T3) hormones. There are multiple reasons why hypothyroidism can develop in an individual, with Hashimoto’s disease being the leading cause. Hashimoto’s disease is an autoimmune type of thyroiditis where the prevalence of antibodies is seen in the patient, attacking and damaging the cells in the thyroid gland in a manner that reduces the quantity of the T4 and T3 hormones significantly, as well as inflaming the thyroid gland [1]. Breast fibroadenomas, commonly occurring in women in their reproductive, pre-menopausal years, are most often painless benign tumors in one or both breasts [2]. Their size is 2.5 cm on average but they can grow up to 5 cm or larger. Moreover, in the majority of cases, they are not associated with a risk of cancer [2]. It is also speculated that Hashimoto’s thyroiditis is associated with breast fibroadenomas. An observational study supported the association between the two conditions [3]. Therefore, studying the coexistence of both diseases, simultaneous manifestation, possible correlation, and the best available methodologies/approaches to treat it is essential. It is also important to encourage physicians to educate female patients about the possibility of benign breast conditions in the presence of Hashimoto’s thyroiditis.

## Case presentation

**Figure 1:**
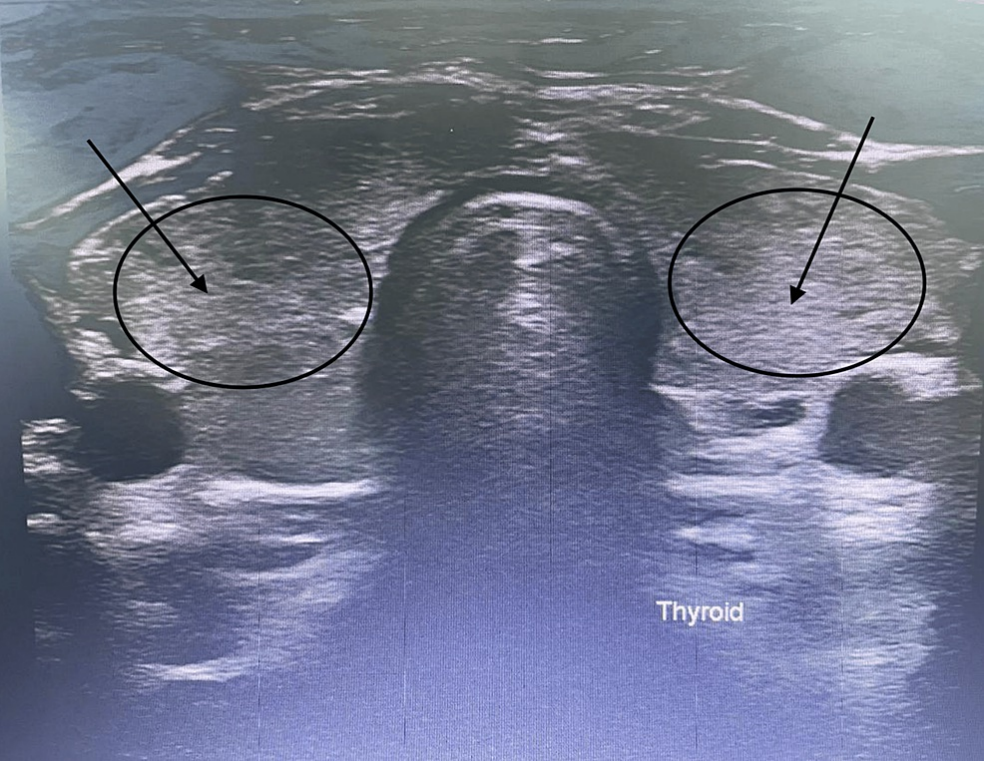
Ultrasound image of the thyroid gland. Heterogenous echogenicity of the thyroid can support hypothyroid findings.

A 34-year-old, married, nulliparous female presented to the endocrinology clinic with the chief complaint of fatigue, which progressed and worsened over a year. She was referred to the endocrinology clinic by the gynecologist that she visited in the same hospital where she was diagnosed with nipple discharge, lower abdominal pain, vitamin D deficiency, weakness, fatigue, and suspected hypothyroidism. The patient had a height of 160 cm and a weight of 63.02 kg (for a body mass index of 24.62 kg/m^2^). On the day of presentation to the endocrinologist, her vitals were within normal ranges. Her menstrual cycle had been normal and uneventful, with no medications for chronic medical conditions. Her family history was unremarkable for the given case but included her father having liver cancer. Her mother and brother were on anti-hypertensive medications due to primary hypertension. Consequently, tests to assess thyroid-stimulating hormone (TSH), serum cortisol, serum creatinine, random blood glucose levels, and potassium levels were ordered, along with ultrasound imaging for the thyroid gland. Creatinine, potassium, cortisol, and blood sugar levels were normal. However, TSH levels were markedly elevated at 10 mIU/L. The ultrasound imaging also showed an average-sized thyroid gland, with both thyroid lobes and the isthmus being of average size showing heterogeneous parenchymal texture with no suspicious nodules or cysts (Figure 1).

The right thyroid lobe measured 47 × 19 × 18 mm along its maximal anteroposterior, transverse, and craniocaudal dimensions, respectively. The left thyroid lobe measured 42 × 18 × 16 mm in its anteroposterior, transverse, and craniocaudal dimensions, respectively. The thyroid isthmus measured 3.5 mm in the anteroposterior dimension.

﻿No sizable enlarged cervical lymph nodes were noted with few bilateral non-specific subcentimeter nodes, with no other remarkable findings in the neck.

Anti-thyroglobulin antibody and anti-thyroperoxidase antibody levels were also obtained from the patient’s blood serum samples, which were also elevated at 126.8 IU/ml (normal = 0-116 IU/mL) and 303.4 IU/mL (normal = 0-34 IU/mL), respectively.

The patient was diagnosed with Hashimoto’s thyroiditis and treated with Euthyrox (levothyroxine) 50 µg/day. The follow-up was scheduled after two months of treatment to re-check the TSH levels which were now normal at 1.1 mIU/L (normal = 0.45-4.12 mIU/L).

Simultaneously the patient was also being monitored by a general and breast surgeon, as she presented to the doctor in the outpatient clinic of the same hospital with breast pain and multiple breast lumps. The right breast showed the following findings: 11 o'clock - 16 × 7 mm, 3 - 13 × 5.5 mm, 3 o'clock - 18 × 7 mm with lobulated margins, and retroareolar - 8 × 5 mm. The left breast showed the following findings: 2-3 o'clock - 11 × 6 mm, 3 o'clock - 13 × 8 mm with lobulated margins, 4 o'clock - 9 × 5.4 mm, and 10 o'clock - 10 × 5.7 mm.

**Figure 2:**
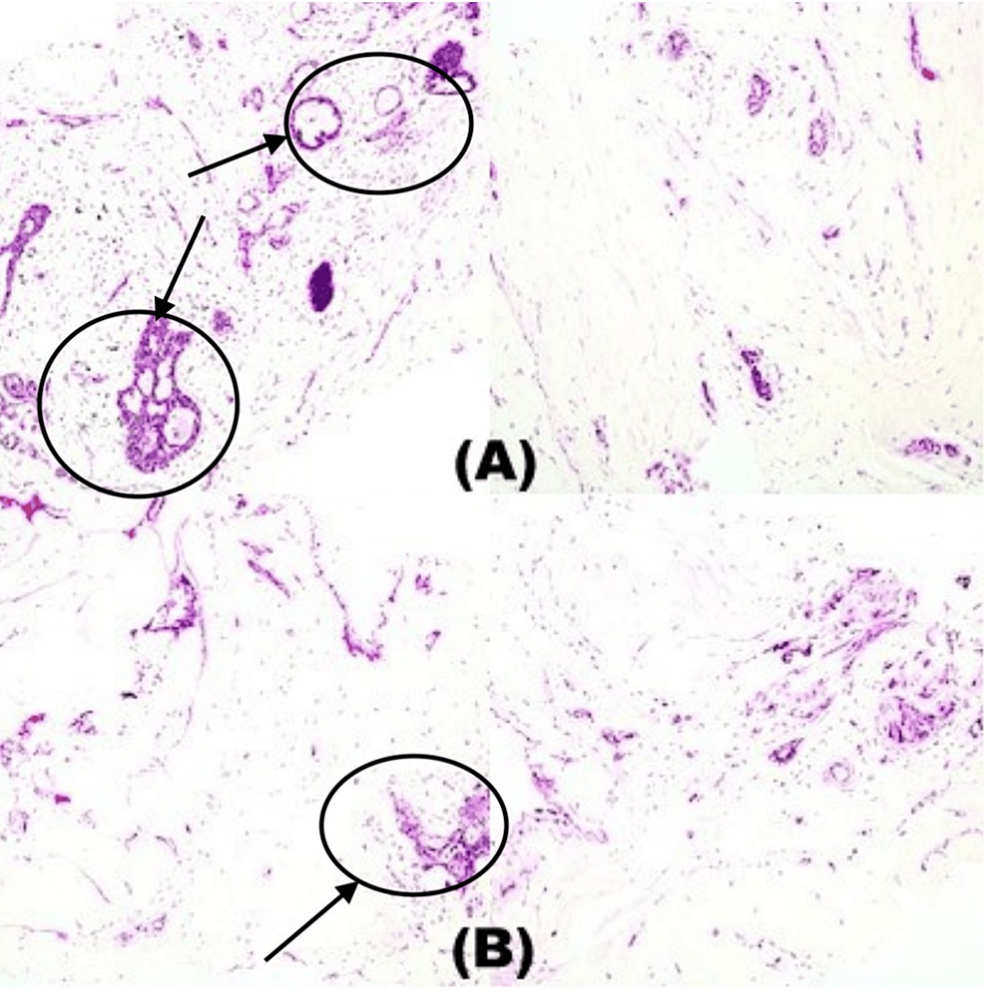
Right (A) and left (B) breast core biopsy findings, respectively. Breast tissue core biopsy showing fibroadenoma with a predominance of intracanalicular pattern showing connective tissue stroma invaginating into the gland spaces and compressing with a siltstone-like appearance. The stroma is composed of loose connective tissue with areas of early myxoid degeneration. Stromal and epithelial hyperplasia is notable, but no stromal or ductal atypia can be seen.

Upon biopsy and histopathological examination ordered by the surgeon, the physical examination revealed multiple mobile and tender lumps in both breasts. She was revealed to have fibroadenoma in both breasts. Breast tissue core biopsy showed breast fibroadenoma with a predominance of intracanalicular pattern showing connective tissue stroma invaginating into gland spaces and compressing with a siltstone-like appearance. The tubules were formed mostly of cuboidal-to-low columnar cells with round nuclei resting on the myoepithelial cell layer. The stroma was made up of loose connective tissue with areas of myxoid degeneration. Stromal and epithelial hyperplasia is notable, but no stromal or ductal atypia was seen (Figure 2).

TSH levels before and after treatment are presented in Table 1.

**Table 1:** TSH levels of the patient before and after treatment with levothyroxine. TSH = thyroid-stimulating hormone

TSH level before treatment	TSH level after treatment
10 mIU/L	1.1 mIU/L

## Discussion

A substantial proportion of the global population suffers from hypothyroidism, roughly 5% in 2019, in particular, Hashimoto’s thyroiditis, whose incidence was 0.8 per 1,000 males and 3.5 per 1,000 women annually in 2022 [4]. Breast fibroadenoma affects 10% of the global female population once in their lifetime [5].

Hashimoto’s can present with an array of symptoms, usually beginning with fatigue/tiredness, weight gain, increased sensitivity to cold temperatures, drying of the skin, and suboptimal cognitive function. Once the symptoms and signs of the patient clinically indicate hypothyroidism, the final diagnosis is confirmed on blood tests and ultrasound imaging to confirm the disease and Hashimoto’s variant. The tests usually involve checking the TSH, T3, and T4 levels for hypothyroidism and anti-thyroid peroxidase and/or anti-thyroglobulin antibodies to confirm Hashimoto’s disease. [1] Ultrasound scans are based on size, echogenicity, and often vascularity of the gland in comparison to the normal thyroid gland as well as the presence of any structural differences such as nodules. The treatment typically involves a synthetic supply of the T4 hormone, such as levothyroxine to maintain the patient’s vital metabolic functions. Accurate calculations of dosing and follow-ups are essential to establish successful maintenance in this condition as the lack of it has a significant impact on the patient’s quality of life and long-term negligence can lead to long-term and often severe health complications, such as cold intolerance, decreased sweating, nerve deafness, peripheral neuropathy, decreased energy, depression, dementia, memory loss, muscle cramps, menorrhagia, and pressure symptoms in the neck from goiter enlargement such as voice hoarseness [1].

This case highlights the intriguing co-occurrence of Hashimoto’s and breast fibroadenomas in a relatively young woman. While both conditions are independently prevalent, their simultaneous presence, one being the causality factor of the other, or any other benign breast disease [1,6] warrants exploration of potential underlying connections. Although a definitive link, i.e., specificity, remains unestablished, two main theories suggest a plausibility.

Regarding hormonal factors, estrogen dominance, a common feature of Hashimoto’s [7,8], could stimulate breast cell proliferation [2,9] and contribute to fibroadenoma development. Regarding immune dysregulation, autoimmune processes underlying Hashimoto’s might also affect breast tissue, influencing fibroadenoma formation. Shared genetic susceptibility linked to both conditions may increase an individual’s risk of developing both Hashimoto’s and fibroadenomas.

Optimal thyroid hormone replacement remains the cornerstone therapy for Hashimoto’s thyroiditis [10], while careful monitoring of serum thyroid function and potential side effects is essential. For fibroadenomas, regular clinical and imaging follow-up is recommended to ensure benignity and detect any changes suggestive of malignancy. Surgical intervention for breast fibroadenomas is rarely indicated unless suspicious features emerge or symptoms become bothersome.

## Conclusions

The coexistence of Hashimoto’s thyroiditis and breast fibroadenomas emphasizes the importance of thorough clinical evaluation and collaborative care between endocrinologists and breast specialists, with current studies highlighting mainly the prevalence. Exploring hypotheses based on hormonal dysregulation, autoimmune overlap, and shared genetic susceptibility may pave the way for future research and improved understanding of this intriguing clinical association. Personalized management of both conditions is the foundation for optimal patient care. We aim to emphasize the coexistence such that all females with Hashimoto’s thyroiditis are considered for the possibility of the presence of benign breast conditions such as fibroadenomas. We also wish to highlight the importance of encouraging the study of the consistency of this correlation as well as suggest the discussion of this simultaneous coexistence in patients diagnosed with Hashimoto’s thyroiditis, with female patients being part of patient education and giving them a foresight of possible manifestations.
